# Mathematical models to characterize the early phase of the COVID-19 pandemic in New Mexico, USA

**DOI:** 10.3934/mbe.2025093

**Published:** 2025-07-29

**Authors:** Annika Vestrand, Gilberto González-Parra

**Affiliations:** Department of Mathematics, New Mexico Tech, New Mexico 87801, USA

**Keywords:** mathematical models, spatio-temporal, COVID-19, county data

## Abstract

In this paper, we used a variety of mathematical models to characterize the early phase of the COVID-19 pandemic in New Mexico. We used both empirical and mechanistic models based on differential equations to examine the dynamics of the pandemic in New Mexico and in carefully selected New Mexico counties. For the empirical model, we used the exponential growth model to compute and estimate the growth rate, basic reproduction number ${ℛ}_{0}$, and effective reproduction number ${ℛ}_{t}$. In addition, we used the SIR model to estimate ${ℛ}_{0}$, using the new weekly COVID cases and also cumulative cases. We found that for the beginning of the early phase of the pandemic, the most populous counties had basic reproduction numbers greater than one. In addition, it was found that the transmission rates of some counties varied significantly during the early phase of the pandemic. Moreover, ${ℛ}_{0}$ dropped below one during some phases for some counties when using the SIR model. This suggests that non-pharmaceutical interventions had some impact on reducing the burden of the pandemic and that people’s behavior changed during this early phase.

## 1. Introduction

The first SARS-CoV-2 case emerged in December 2019, and later the WHO declared the COVID-19 pandemic. This pandemic has caused devastating worldwide effects. In January 2025, SARS-CoV-2 has claimed 7.1 million lives around the world [1]. In particular, in the United States, there have been more than one million deaths. In the state of New Mexico, there have been almost 10,000 deaths and more than 700k cases during the COVID-19 pandemic [1]. New Mexico has a population of approximately 2.1 million. New Mexico is located in the southwest U.S. and has a very particular population compared to the rest of the country [2]. New Mexico is ranked as the second highest state by percent of the population identifying as American Indian or Alaska Native [2]. A large part of this population lives in the northwest region of the state. Thus, all these factors make the study of the COVID-19 pandemic interesting. In particular, the early phase since this period was less affected by external factors due to the initial spread of SARS-CoV-2 and the travel restrictions implemented in some U.S. states.

The basic reproduction number ${ℛ}_{0}$ is a very important number that has been extensively used to characterize different epidemics and pandemics [3, 4]. This number represents the number of secondary infections caused by a single infected person in a population of only susceptible individuals [3]. Thus, this number provides information on how the epidemic is growing. A larger ${ℛ}_{0}$ means that the virus can spread to many people and poses a high risk to public health. Therefore, computing and estimating ${ℛ}_{0}$ is very useful in understanding the dynamics of epidemics and pandemics. Another aspect related to ${ℛ}_{0}$ is that, for instance, an increase in ${ℛ}_{0}$ by 0.4 can enhance the transmissibility of the disease by 50–70% with a four-fold increase in viral load [5, 6]. It is important to mention that the basic reproduction number ${ℛ}_{0}$ depends on the transmission rate of the virus, which depends on the contact rate between people, and it is affected by social factors [3]. Furthermore, social factors have evolutionary implications that can cause host heterogeneity. In [7], it was shown that a disease control selects for asymptomatic strains. The basic reproduction number ${ℛ}_{0}$ is given by a function of some of the parameters of the particular model [3]. For example, in the SIR model, the transmission rate is the product of the average number of contacts per person per time and the probability of virus transmission in a contact between a susceptible person and an infected one [3, 8].

Another important metric is the time-varying effective reproduction number ${ℛ}_{t}$ [9–13]. This number ${ℛ}_{t}$ includes the effect of time-varying factors on the spread of a disease, where not all of the population is susceptible. Therefore, it can be used at any time during the epidemic, where an unknown fraction of the population can be infected. The time-varying effective reproduction number ${ℛ}_{t}$ represents the average number of secondary infections that an infected individual is expected to produce at some particular time. Thus, this versatile metric is also useful in understanding the dynamics of epidemics and pandemics. Another approach to assess the potential damage of an epidemic is by using the growth rate in the early phase of the epidemic [14–16]. Using the exponential growth rate model, it is possible to estimate the basic reproduction number ${ℛ}_{0}$ by means of the value of the first generation interval and the growth rate [4, 17]. Similarly, the logistic growth rate model can also be used to estimate the basic reproduction number ${ℛ}_{0}$.

Previous works have used mathematical models to compute the basic reproduction number ${ℛ}_{0}$ or the effective reproduction number ${ℛ}_{t}$ during the COVID-19 pandemic. Many of these models are based on differential equations [18–21]. In [22], the exponential growth rate and the basic reproduction number ${ℛ}_{0}$ of COVID-19 were estimated using the Poisson likelihood framework. In [23], an SEIR-type model was fitted to the data to estimate ${ℛ}_{0}$ in different states of the USA. In [24], the SIR model was used to estimate the basic reproduction number ${ℛ}_{0}$ in Costa Rica. It was found that for the first six days, ${ℛ}_{0}$ was approximately 2.58. Then, after the implementation of non-pharmaceutical interventions (NPIs) ${ℛ}_{0}$ dropped to 1.12. In [25], the effective reproduction number for five counties in California was estimated using wastewater data. In [26], a proposed model was fitted to COVID daily data from the United Kingdom (UK) and a basic reproduction number of 1.7291 was computed. In [22], an exponential growth rate was estimated as 0.22 per day and the basic reproduction number, ${ℛ}_{0}$, as 2.37. In [27], the basic reproduction number was estimated for each district of India. The cumulative number of cases of COVID-19 was used to calibrate the exponential growth model for the first 7–15 days of the outbreak. It was found that ${ℛ}_{0}$ was as large as 7 in a few districts. In [28], the logistic growth model was calibrated using the new daily cases of COVID-19 from eleven selected Chinese provinces and different municipalities in South Korea and Iran. The growth rates were found to be different between the provinces of China and between South Korea and Iran. In [29], an SEIR-type model was calibrated using daily COVID-19 data for five provinces of India. In [30], a mathematical model for the transmission dynamics of SARS-CoV-2 was proposed where the parameters are piecewise constant functions. The idea of this approach is to introduce changes in human behavior and the impact of public health policies on the dynamics of the COVID-19 pandemic. In addition, the model was applied to consider the real data from COVID-19 in Portugal, since March 2, 2020, until September 17, 2020. Besides all these works that have used mathematical models, there are other types of models that have been used for COVID-19 prognosis prediction [31, 32].

In this paper, our objective is to use a variety of mathematical models to characterize the early phase of the COVID-19 pandemic in New Mexico. We will use empirical and mechanistic models based on differential equations to examine the dynamics of the pandemic in carefully selected New Mexico counties. For the empirical model, we use the exponential growth model to compute and estimate the growth rate and the basic reproduction number, ${ℛ}_{0}$. With regard to the mechanistic model, we aim to use the SIR model. All these mathematical models have been used extensively to compute the basic reproduction number ${ℛ}_{0}$. We also aim to investigate the impact of the implementation of non-pharmaceutical interventions (NPIs) in New Mexico, USA. We chose New Mexico because of its unique features and the fact that the state had strict NPIs. To the best of our knowledge, mathematical models have not yet been used to characterize and explain the crucial early phase of the COVID-19 pandemic in New Mexico. Thus, this work provides insights into the dynamics of the COVID-19 pandemic that can be useful for public health and can offer advice for future epidemics.

The organization of this paper is the following. In Section 2, we present the data for the early phase of the COVID-19 pandemic in New Mexico, USA. In Section 3, we present the mathematical models that are used to characterize the early phase of the COVID-19 pandemic in New Mexico. Section 4 is devoted to the presentation of the results of the characterization of the early phase of the COVID-19 pandemic in New Mexico. Finally, in Sections 5 and 6, discussions and conclusions are presented.

## 2. Data of the early phase of the COVID-19 pandemic in New Mexico, USA

In this section, we present the data for the early phase of the COVID-19 pandemic in New Mexico, USA. In particular, we present the data by counties.

### 2.1. Early phase of the COVID-19 pandemic in New Mexico

**Figure 1.**
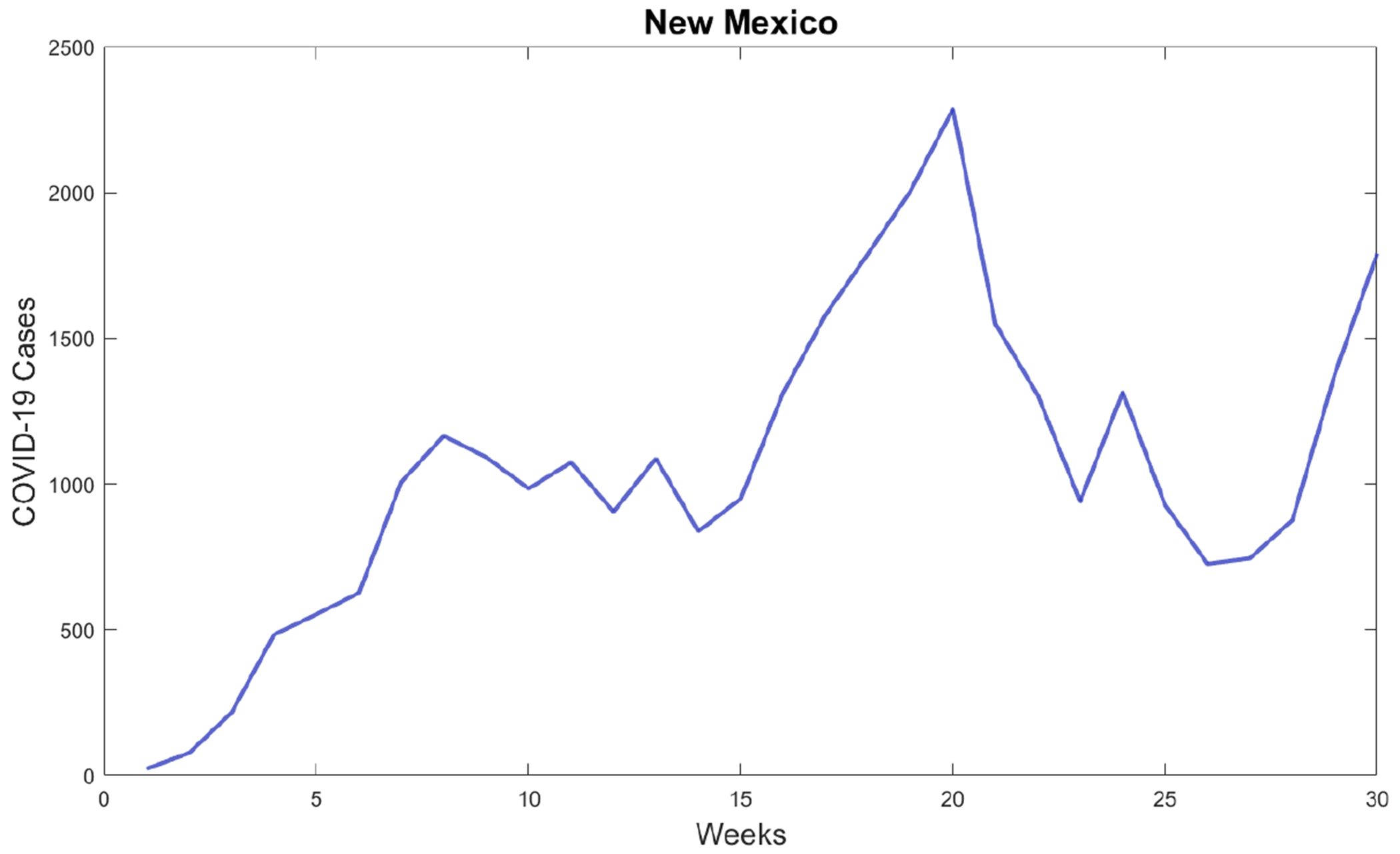
Weekly confirmed COVID-19 cases for the first 30 weeks of the COVID-19 pandemic in the state of New Mexico, USA.

**Figure 2.**
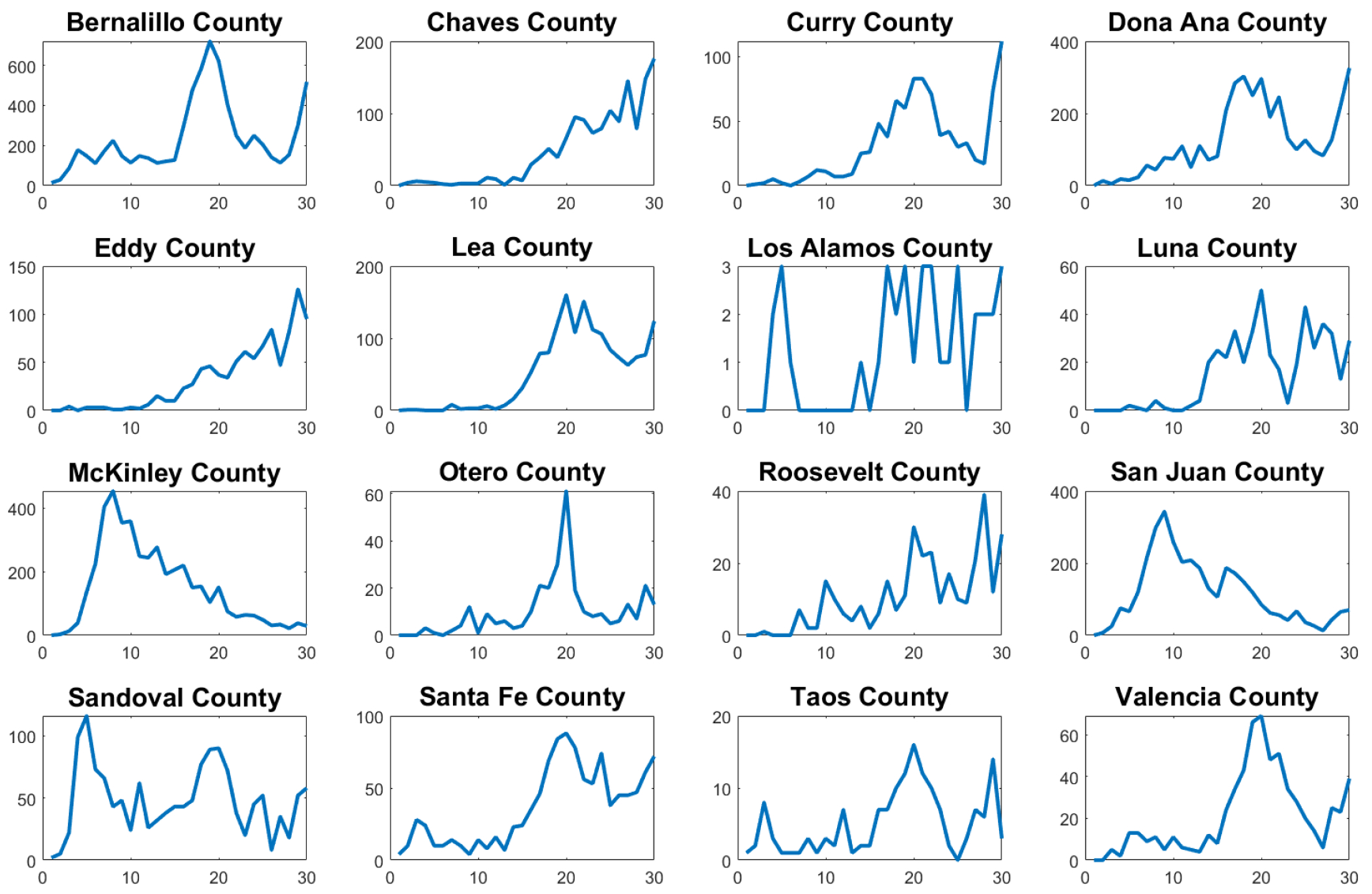
Weekly confirmed COVID-19 cases in New Mexico’s 16 most densely populated counties for the first 30 weeks of the COVID-19 pandemic.

**Figure 3.**
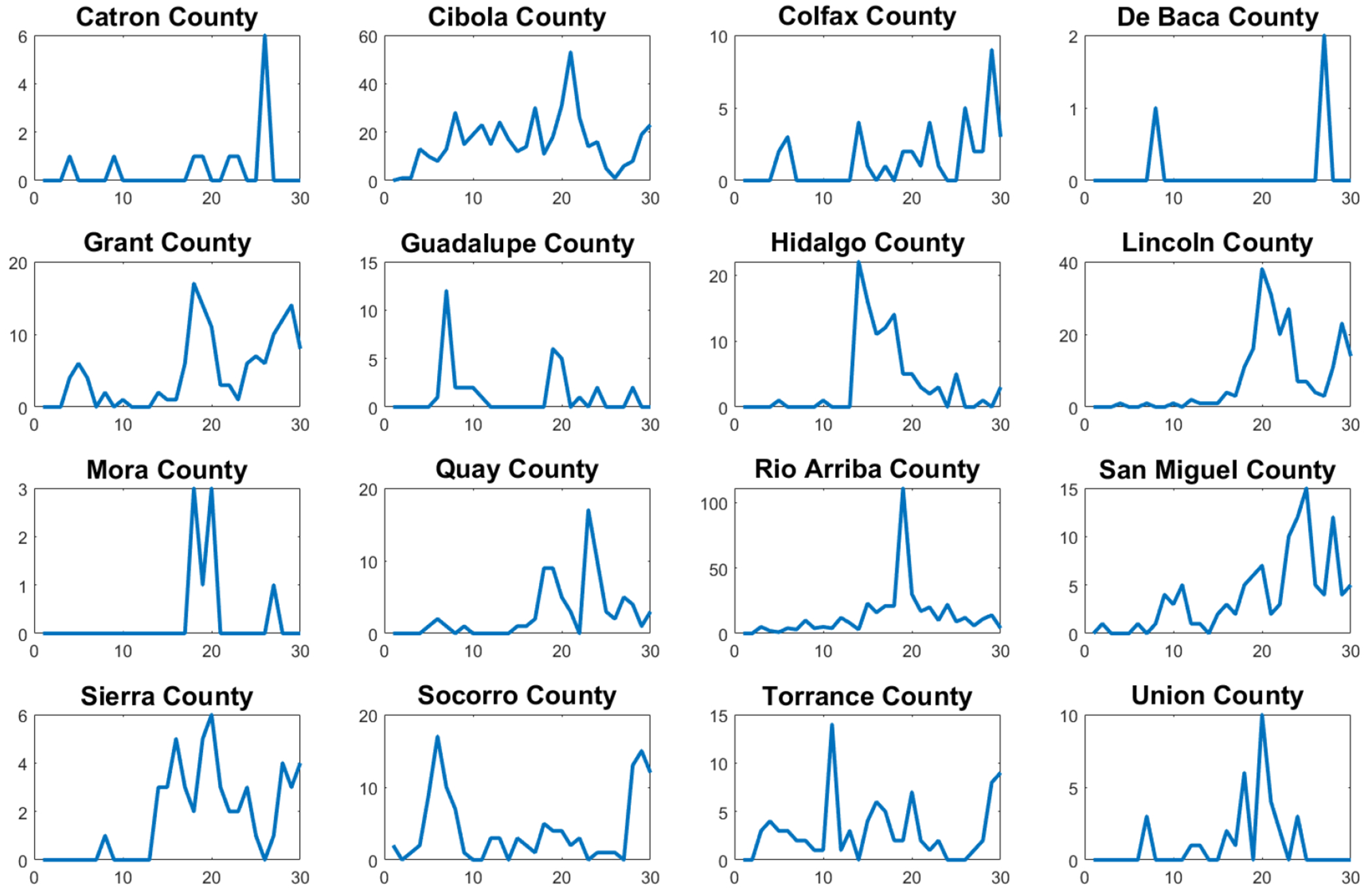
Weekly confirmed COVID-19 cases in New Mexico’s 16 least densely populated counties (excluding Harding County) for the first 30 weeks of the COVID-19 pandemic.

The first wave of the COVID-19 pandemic lasted from mid-March to late June in New Mexico. COVID-19 cases were first reported in New Mexico on March 11, 2020, and continued to increase until early May 2020, after which the cases stabilized until the second wave began in late June 2020. This second wave lasted until August 2020. Figure 1 shows the new confirmed cases for the whole state of New Mexico. This data related to new confirmed cases was reported in the John Hopkins COVID-19 data set [33]. The first week of data represents the week of March 10–17. The pattern of New Mexico’s state is not shared by all New Mexico counties, as can be seen in Figures 2 and 3. Many counties did not experience a clear first wave during the time the first wave occurred across the state, and some counties showed quite different behavior. In particular, McKinley County experienced a first wave that lasted from March until November, which spanned the entire time of the first two waves of the whole state. It is important to mention that McKinley County has San Juan and Sandoval as adjacent counties. San Juan County showed a similar pattern, but did experience a noticeable increase in cases around the end of June that corresponds to the statewide second wave. During the first 10 weeks of the COVID-19 pandemic, these counties had the highest per capita case rates in New Mexico, and McKinley County remained the highest in the state until the statewide second wave began. With regard to Sandoval County, the pattern of the early phase of the COVID-19 pandemic was different from that of McKinley County and San Juan counties. It is important to mention that Sandoval County is adjacent to Bernalillo County, which is the most populous county in New Mexico. Thus, the mobility of people could have affected the spread of SARS-CoV-2 in a different way than McKinley and San Juan counties. In addition, McKinley and San Juan counties have large areas that are part of the Navajo Nation. The prevalence of COVID cases in the Navajo Nation was among the highest in the United States. There was a significant increase in the number of cases during the early phase of the COVID-19 pandemic with a doubling time of 10.12 days [34]. Therefore, these facts could have affected the dynamics of the early phase of the COVID-19 pandemic in these counties.

Many counties in the state of New Mexico also showed multiple small peaks in cases that lasted for short periods. These may have been the result of NPIs, as many public policy changes were enacted during the early phase of the pandemic in New Mexico [35].

### 2.2. Selection of counties for characterization

We chose to examine the early COVID-19 pandemic in New Mexico’s 16 most densely populated counties with one exception. Rio Arriba County was examined instead of Los Alamos County, the second most densely populated county in New Mexico, due to the fact that its case data showed an extremely high number of irregularities that made finding meaningful fits impossible. In addition, we choose to focus on the most densely populated counties because one of the models we used to examine the COVID-19 pandemic, the SIR model, makes the assumption of homogeneous mixing [3]. We believe that this condition is unlikely to be met in the less densely populated counties as random encounters between individuals are less likely to occur. In addition to this, many of the less densely populated counties have very small populations below 20,000, which led to a high number of irregularities in the incidence case data, making them poor candidates for fitting with the mathematical models. For small populations, stochastic models might be more appropriate [36, 37]. For example, in [38], a stochastic epidemiological model was presented as an extension of a compartmental SEIR model to analyze the dynamics of the COVID-19 pandemic in the city of Bogotá, Colombia. This model incorporated the transmission of COVID-19 impacted by social behaviors and considered mitigation measures, such as confinement and partial relaxed restrictions. Thus, the transmission rate was changed according to the dynamics of the confinement or lockdown policies. This process of changing the transmission rate depending on the NPIs is challenging due to the variety of factors that affect the transmission rate. In the next section, we will present the mathematical models that will be used to characterize the early phase of the COVID-19 pandemic in New Mexico.

## 3. Mathematical models used to characterize the early phase of the COVID-19 pandemic in New Mexico

In this section, we present the different mathematical models that are used to characterize the early phase of the COVID-19 pandemic in New Mexico. We use two classical epidemiological mathematical models. The first one is the exponential growth model and the second one is the well-known SIR model. Many other works just use the SIR model to characterize epidemics [10, 39–43]. One main aim of this work is to characterize the spatial variability and the effect of NPIs. The SIR model is more appropriate than the exponential model with regard to this aim due to the fact that the exponential model is more suitable for the early phase of epidemics [44]. Other models that have been used to fit to the early phases of an epidemic are the generalized exponential model, the Richards model, the generalized Richards model, and the Gompertz model [19, 45–47].

### 3.1. Exponential growth model

The exponential growth model can be used to look at the early period of an epidemic in which cases increase [4, 15, 16, 47]. However, the model is limited as it can only capture the growth phase of the epidemic and not its later phase, where cases begin to decrease and the virus is brought under control. Moreover, the model does not offer an explanation for the underlying biological process under the SARS-CoV-2 spreads, that is, it is an empirical model [47]. Despite this limitation, if the model is used to examine multiple regions, it can still be used to compare the severity of the epidemic and the effectiveness of public health responses across the regions examined during the early part of the epidemic [4, 15, 47, 48]. The exponential growth model is given by the following equation [47],

(3.1) $$I^{\prime }(t)=rI(t),$$

where r is the exponential growth rate and t represents time. The solution to this ordinary differential equation (ODE) is given by

(3.2) $$I\left(t\right)=I_{0}e^{rt},$$

where $I_{0}$ is the initial number of cases at t=0. Although the exponential growth rate itself can be used to compare epidemics between regions, if the recovery rate of the disease γ is known, it can be used to find the transmission rate of the disease. Recall that at the beginning of the epidemic the susceptible population makes up almost the whole population. Thus, if we use the SIR model (without demographic factors and standard incidence), one gets that $I^{\prime }(t)=\beta I(t)-\gamma I(t)=(\beta -\gamma )I(t)$. Then, it can be deduced from Eq (3.1) that r=β−γ. Thus, we can compute the transmission rate by using the following relationship [49]:

(3.3) β=r+γ.

Then for the SIR model, we can compute the basic reproduction number ${ℛ}_{0}$ since, for the SIR model without demographics, ${ℛ}_{0}=\beta /\gamma$ (for more details, see [3, 49]).

### 3.2. SIR model

In this subsection, we briefly present the well-known mechanistic SIR model [3]. This model is one of the simplest epidemiological models based on a system of differential equations. There are many models that are derived from this one. The model divides the population into three classes: S, susceptible individuals, I, infectious individuals, and R, recovered individuals. The SIR model is given by

(3.4) $$S^{\prime }=-\beta S\;I/N,\;I^{\prime }=\beta S\;I/N-\gamma I,\;R^{\prime }=\gamma I,\;C^{\prime }=\beta S\;I/N,$$

where C is an auxiliary variable that represents the cumulative infected cases [3, 47]. This SIR model fitted to epidemiological data allows us to estimate the transmission rate β if the parameter γ is known. It also allows to estimate the basic reproduction number ${ℛ}_{0}$ [3]. There are a variety of epidemiological models related to the SIR model. For instance, removing N from system (3.4) provides a similar SIR model where the assumption of the underlying transmission process is different (for more details, see [3]).

## 4. Results of the characterization of the early phase of the COVID-19 pandemic in New Mexico

In this section, we present the results of the characterization of the early phase of the COVID-19 pandemic in New Mexico. We use the mathematical models presented in the previous section. We use the Sum of Squared Residuals (SSR) or Residual Sum of Squares (RSS) metrics as the objective function to minimize. There are many other metrics, but the SSR is a classical one that has good statistical properties [50]. It is important to mention that we do not use the SSR to compare the mathematical models, instead we use it to estimate the parameters. The parameters that we estimate of the exponential growth model and the SIR model are identifiable with the available data [45]. Thus, we obtain a unique set of parameters for each model. Nevertheless, assuming that the data have errors, we compute confidence intervals for the estimated parameters by using the bootstrapping method [16, 47]. The bootstrapping method has been used in previous works related to the mathematical modeling of the COVID-19 pandemic with NPIs [51].

### 4.1. Exponential growth model

To compare the response to the COVID-19 pandemic in New Mexico at the county level, we estimated each county’s exponential growth rate and the basic reproduction number ${ℛ}_{0}$ by calibrating the exponential growth model to the data set of confirmed cases of COVID-19 [33]. Epidemiological data are transformed to weeks to smooth the data. The model is calibrated using built-in numerical functions from MATLAB. To find the estimated parameters, we use MATLAB’s *fminsearch* built-in function to minimize the SSR using the objective function found in Eq (4.1). The basic reproduction number ${ℛ}_{0}$ is then calculated from the estimated parameter r using Eq (3.3). The optimization problem is the following:

(4.1) $$\underset{r}{\text{min}}\;SSR=\underset{r}{\text{min}}\underset{j}{\sum }{\left({\hat{I}}_{j}-I_{0}e^{rt_{j}}\right)}^{2},$$

where $t_{j}$ denotes time at week j, ${\hat{I}}_{j}$ denotes new cases for week j,r is the exponential growth rate, and $I_{0}$ represents the initial infected cases [49]. The exponential growth model is sensitive to the start time and the fits always underestimate the true exponential growth rate since they do not take into account the slowing of the exponential growth as the epidemic advances [49]. Thus, the fit window affects the estimation of the growth rate. In this section, we use two different windows: one for the first wave and one for the second wave.

The model is calibrated using only case data for the early period of the pandemic when infected cases were increasing, so the data were used until the incidence of cases reached their highest values, the peak of the first wave [49]. However, determining this point is complicated by the fact that spatial effects led to the first wave occurring at different times in different counties and the fact that many counties had irregularities in their data. Looking at the state-wide data, the first peak started in early March and ended in late June, which corresponds to week 1 through 15 in the data, while the second peak ran from late June to August, weeks 15 to 25 of the data. This pattern does not hold true at the county level, with some counties lacking a clear first wave during the state-wide period, while others have a first wave that goes through the first 25 weeks that cannot be easily separated into two distinct waves. This is further complicated by the fact that the first wave has a very short duration of 6 or fewer weeks in some of the counties and never reached higher than 20 new cases a week in others, making it difficult to determine what is meaningful and what is due to irregularities in the data. To try and counteract this, we carried out two sets of fits for the exponential model. One focused on the very early phase of the pandemic and looked at only the first visible peak. The other looked at the second peak within the first 25 weeks, taking into account the later response to the pandemic so that better spatial and temporal comparisons can be made. In summary, the first wave was defined as the first peak in the data, and the second wave was defined as the second peak in the data or the highest point within the first 25 weeks of the data when no clear second wave occurred. For the second wave fits, the two cutoff criteria were used to try to deal with the fact that, for two of the counties, there was only one long wave while the statewide first and second waves occurred, and in others, the first and second waves were not distinct. Notable were the two counties that only had one wave, the two counties with high Navajo nation populations, Mckinley and San Juan, which show relatively different behavior from the other counties.

**Figure 4.**
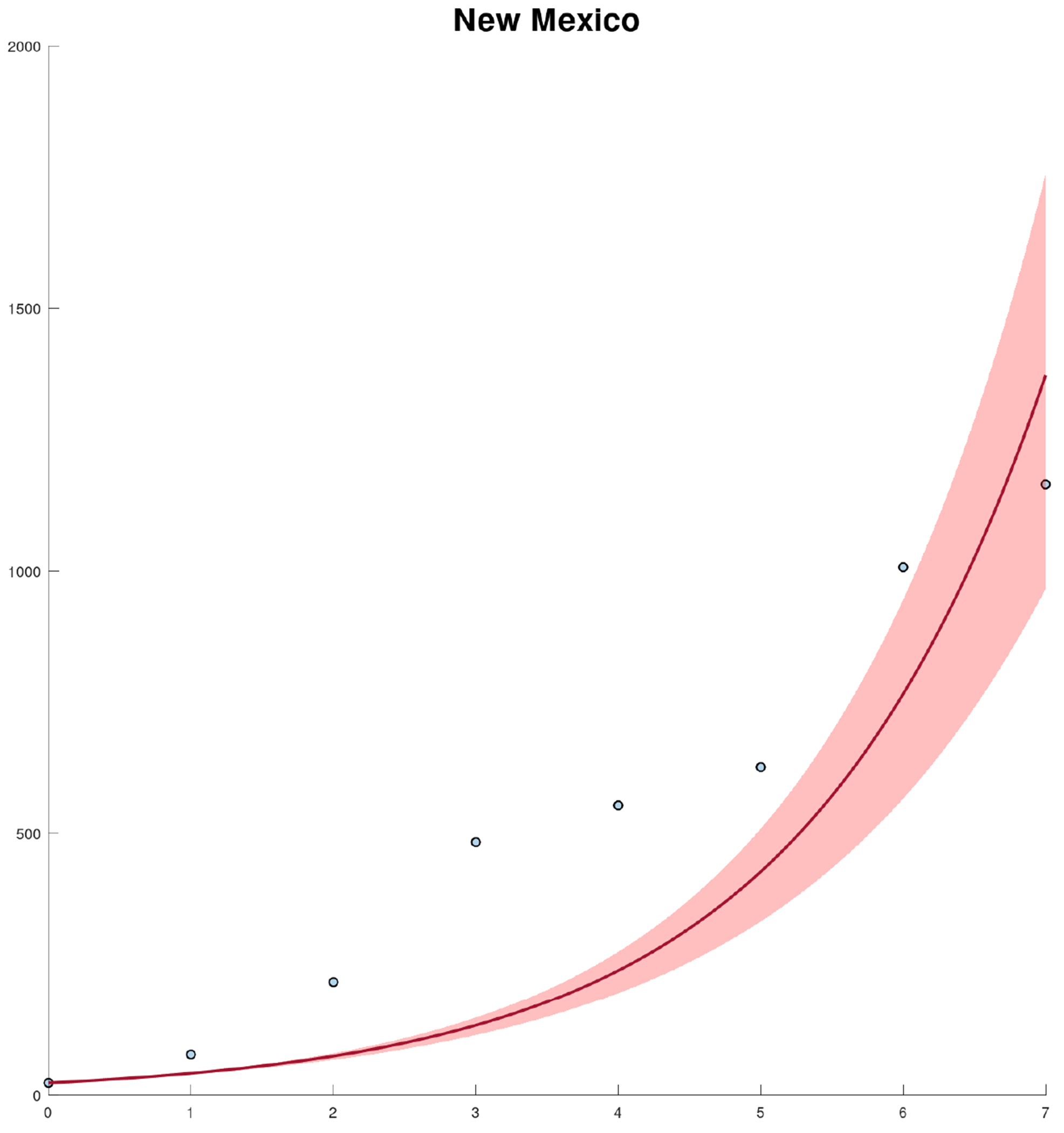
Fits of the exponential growth model (3.1) to the reported cases in New Mexico state for the first weeks of the COVID-19 pandemic. The graph shows the confirmed case incidence (dots), the fitted model (line), and the confidence intervals (shaded region).

**Figure 5.**
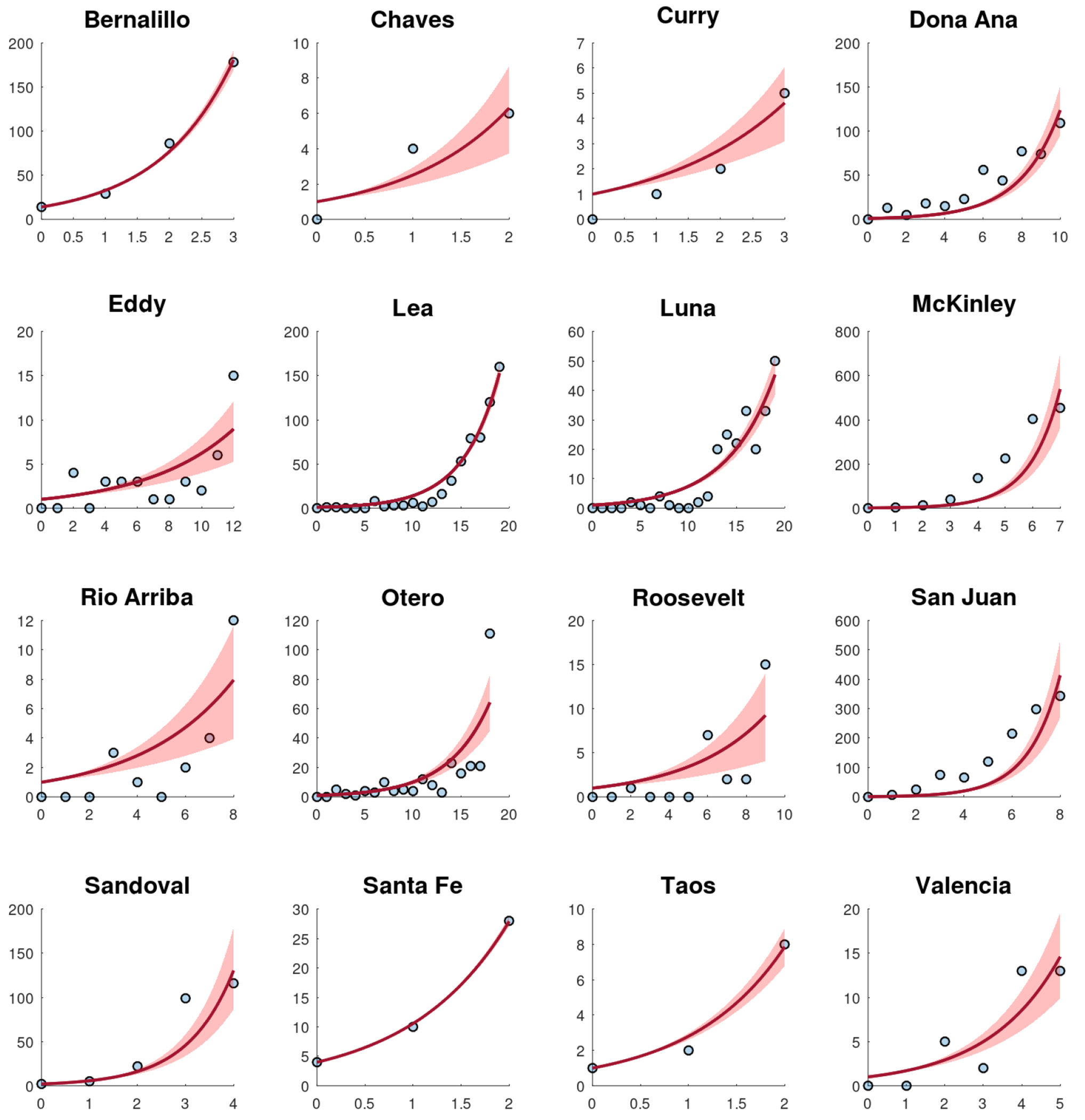
Fits of the exponential growth model (3.1) to the reported cases for some counties for the first wave (peak) of the COVID-19 pandemic. The graphs show the confirmed cases per week (dots) and the fitted model (lines).

Figure 4 shows the calibration of the exponential growth model (3.1) to the first wave of the COVID-19 pandemic in New Mexico. The fit is not very accurate, especially in the middle of the first wave. This could be due to spatial-temporal factors of the COVID-19 pandemic in New Mexico and also to its low population density [52–54]. Therefore, it seems reasonable to calibrate the mathematical models to each county instead of to the data of the whole state. Figure 5 shows the calibration of the exponential growth model (3.1) to the first wave of New Mexico counties with more cases. The fits look relatively well, despite that each county has different features such as the population density and their boundaries with other states and counties. Performing individual fits for each county allows us to characterize the spatial and temporal dynamics of the COVID-19 pandemic by county. This is relevant since the particular characteristics of each county can be inferred and insight can be gained into the COVID-19 pandemic.

**Figure 6.**
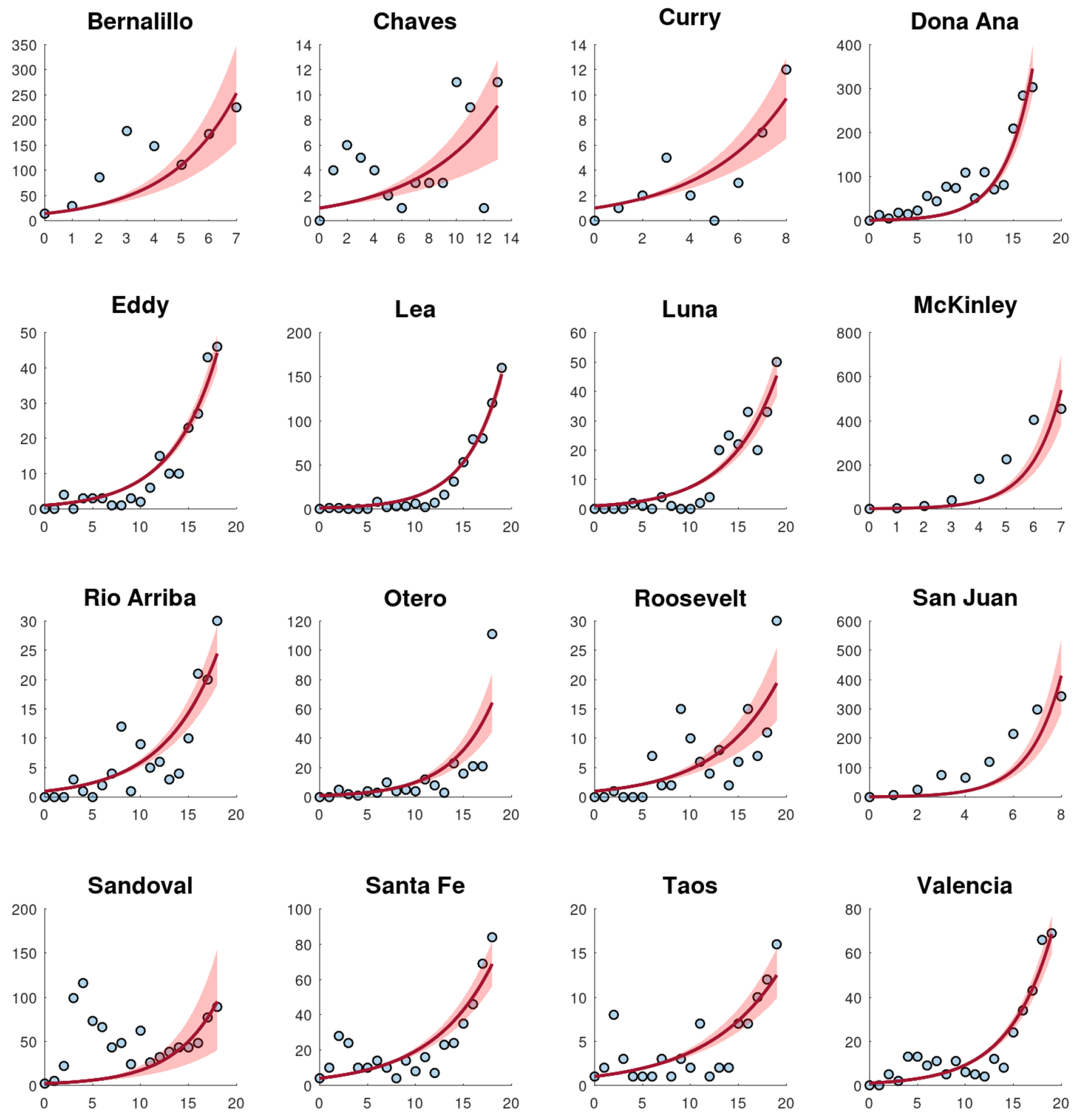
The calibration of the exponential growth model (3.1) to the first wave over the first 25 weeks for New Mexico counties with more cases. The graphs show the confirmed cases per week (dots) and the fitted model (lines).

Figure 6 shows the calibration of the exponential growth model (3.1) to the first wave over the first 25 weeks for New Mexico counties with more cases. In general, most of the fits look relatively well. Some counties show a very early peak of the first wave, which could be due to the effectiveness of the implementation of NPIs [35, 55–57]. A notable case is Bernalillo County, which is one of the most populous counties in New Mexico. In this county, the first peak occurred in the 4th week. However, San Juan County had its first peak much later in the 9th week. Again, several spatial-temporal factors can play a role in these patterns. Moreover, the geographic location of the counties can also be relevant.

Table 1 shows the results of the calibration of the exponential growth model (3.1) for the first wave (peak) during the early phase of the COVID-19 pandemic. In particular, it shows the estimated parameters, the estimated basic reproduction number ${ℛ}_{0}$ and the minimized SSR. It can be seen that for all counties, we get ${ℛ}_{0}>1$. This agrees with the fact that the COVID-19 pandemic was expanding during the early phase of the pandemic. This is despite the implementation of NPIs in New Mexico. The counties with the largest basic reproduction number ${ℛ}_{0}$, are Bernalillo, Chaves, McKinley, Sandoval, Santa Fe, and Taos. Each of these counties except Taos are counties with large populations in New Mexico. In these counties, the spread of SARS-CoV-2 is more likely. Thus, the results are plausible despite all the spatial-temporal factors affecting the pandemic. It is important to mention that with the available data, the parameter r of the exponential growth model (a particular case of the generalized growth model) is identifiable [45]. We performed the bootstrapping method (with Gaussian error structure) to obtain confidence intervals for the parameter r [16, 58]. Table 1 shows the confidence intervals. This provides insight into the characterization of the COVID-19 pandemic in the state of New Mexico and its counties.

Table 2 shows the results of the calibration of the exponential growth model (3.1) for the second and often highest peak over the first 25 weeks. In some cases, the data have a second noticeable peak, which is much higher for some counties and still took place at the time of the statewide first wave for some of the counties. We performed the bootstrapping method (with Gaussian error structure) to obtain confidence intervals for the parameter r [16, 58]. We again obtained that for all counties, ${ℛ}_{0}>1$. These results agree with those obtained for the first few weeks, despite some counties having an early peak during the first few weeks of the pandemic. The counties with the largest basic reproduction number ${ℛ}_{0}$ are Bernalillo, Dona Ana, McKinley, and San Juan. All of these counties have large populations relative to New Mexico. Notice that Taos County is no longer among the counties with the highest basic reproduction number ${ℛ}_{0}$. This is expected due to its small population. Santa Fe County is another county where the fit for the first 25 weeks is no longer among the highest despite the fact that it is the 3rd most populated county in New Mexico. One possible explanation is that the capital of New Mexico is in this county and people in this county might have taken the NPIs more strictly. On the other hand, San Juan County now appears among the counties with the largest basic reproduction number ${ℛ}_{0}$. This county is the 5th most populated county in New Mexico, so the result seems plausible.

### 4.2. SIR

For the SIR model (3.4), we calibrate the model using the cases of COVID-19. First we fit the SIR model to cumulative cases and then we fit it to new cases. Both approaches have been implemented extensively in many works [44, 49]. When using cumulative data the points are correlated, so oftentimes incidence data is used [49]. Nevertheless, some works used parametric bootstrapping to address this drawback [44, 49]. In the SIR model, we estimate the transmission rate β and the basic reproduction number ${ℛ}_{0}$. Although we initially calibrated the SIR model to confirmed cases and scaled the infected cases to account for unreported cases, we found that this method produced unrealistic results. Despite the fact that we obtained reasonable values for β and ${ℛ}_{0}$, the fits found using this calibration predicted that a very large proportion of the population would have been infected with COVID-19 during the first wave, in some cases greater than 80%. These results implied an infection fatality ratio (IFR) of at least 10 times lower than that reported by other sources [59]. For example, in [60] it was estimated that the infection fatality rate in the United States during the early pandemic was 0.863 %. This result was based on serological studies. Thus, based on these preliminary results, we determined that this model was invalid for examining the state of New Mexico at the county level. To counteract this, we chose instead to calibrate our model according to the number of deaths divided by the IFR reported by other works [60]. Then, we used this result as an estimate for the number of true cases and then minimized the SSR generated between the SIR model (3.4) and the aforementioned true cases. Thus, to estimate the true cases we used the real data of deaths and divided them by the IFR. Then, we computed an estimate of the true cumulative cases [49]. There is uncertainty in the number of infected cases due to many factors such as undereporting, asymptomatic cases, etc. [61–64]. Before estimating the true cases, we performed a correlation test between the time series of the number of cases and deaths. We found that the highest correlation occurs with a lag of three weeks between both time series. This result agrees with the scientific literature related to COVID-19 data [65, 66]. Thus, we also attempted to compute the true cases using the number of deaths and the IFR, with a lag of three weeks. However, we found that results were similar but a little bit better using no lag. Therefore, we only present the results with no lag.

Due to the relatively small size of the first wave and the low number of deaths in New Mexico, this limited us to only examining four counties that had particularly large first waves. We also chose to use multiple transmission rates for β since the graphs of the weekly time series show multiple peaks indicating different phases of the COVID-19 pandemic in New Mexico. The state of New Mexico enacted many policy changes during the early phase of the pandemic that impacted the spread of the virus, making it impossible to capture the early dynamics of the pandemic in the state with a timeinvariant transmission rate β. Since local implementation of policy changes and their adoption occurred at different times, we used different phases for each county [35]. A similar mathematical modeling approach using phases was used for the COVID-19 pandemic and influenza [67–72]. The different phases that we used for each county can be found in Table 3. We estimated the parameter β when using new cases (per week) by using MATLAB’s *fminsearch* to minimize the following objective function:

(4.2) $$\underset{\beta_{i}}{min}\;SSR=\underset{\beta_{i}}{min}\;\sum_{j}{\left({\overset{ˆ}{I}}_{j}-D_{j}/IFR\right)}^{2},$$

where $D_{j}$ is the number of deaths that occurred in week j and ${\overset{ˆ}{I}}_{j}$ is the new cases of week j generated by the SIR model. On the other hand, we estimated the parameter β when using cumulative cases by minimizing the following objective function:

(4.3) $$\underset{\beta_{i}}{min}\;SSR=\underset{\beta_{i}}{min}\;\sum_{j}{\left({\overset{ˆ}{C}}_{j}-{DC}_{j}/IFR\right)}^{2},$$

where ${DC}_{j}$ is the number of cumulative deaths until week j and ${\overset{ˆ}{C}}_{j}$ is the new cases of week j generated by the SIR model. It is important to mention that with the available data the transmission rate β of the SIR model is identifiable [45]. We performed bootstrapping to obtain confidence intervals for this parameter.

#### 4.2.1. Fits of the SIR model to cumulative COVID cases

In this section, we present the fits of the SIR model to the cumulative cases of COVID using different phases and transmission rates due to the NPIs. For instance, the mass gathering ban was expanded to include houses of worship on April 11th, 2020. Another example is that the governor announced limited reopening for dine-in restaurants, indoor malls, gyms, salons, and more on May 28th, 2020 [35].

**Figure 7.**
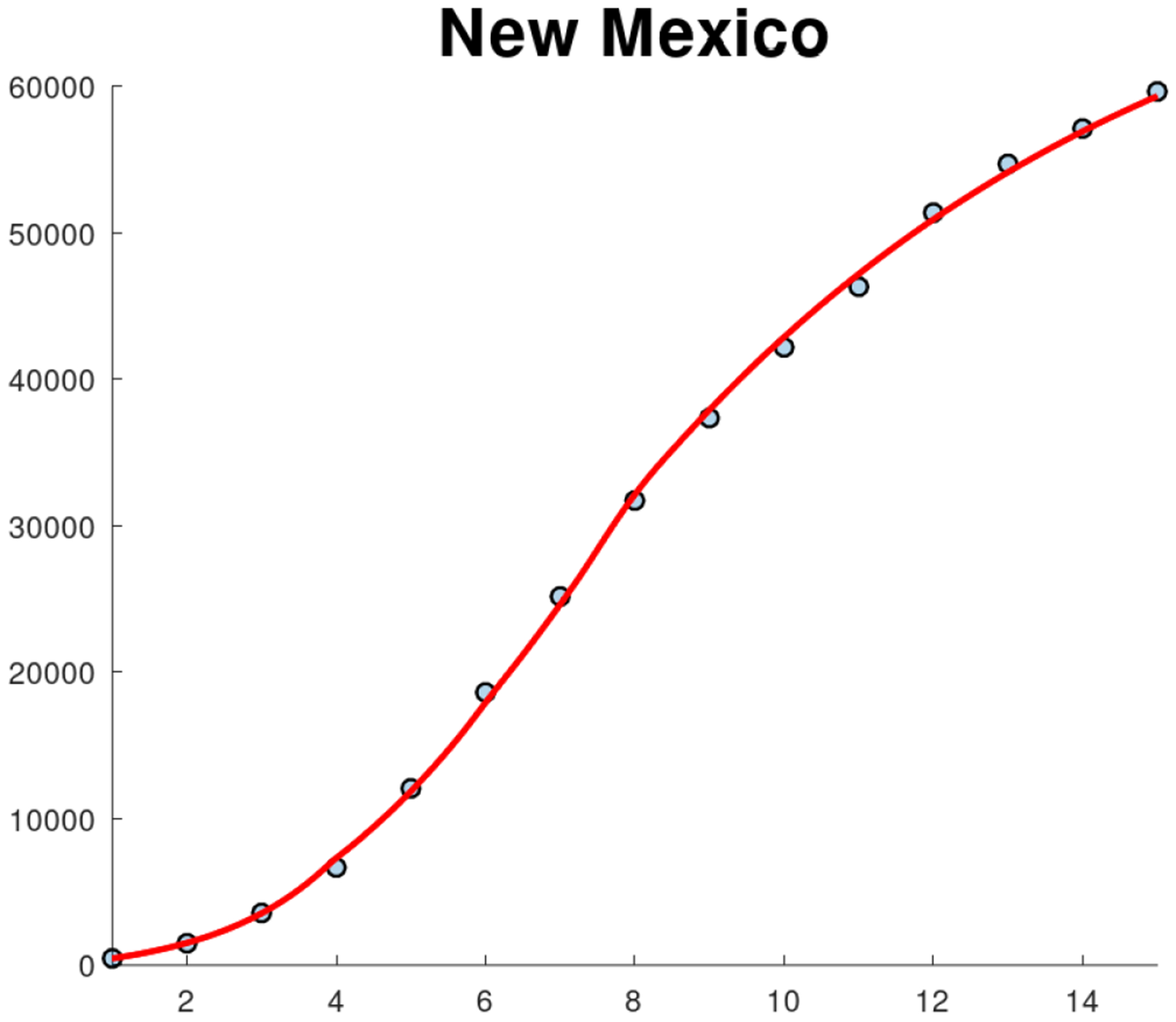
Fits of the SIR model to the cumulative cases of COVID in New Mexico state using different New Mexico phases.

**Figure 8.**
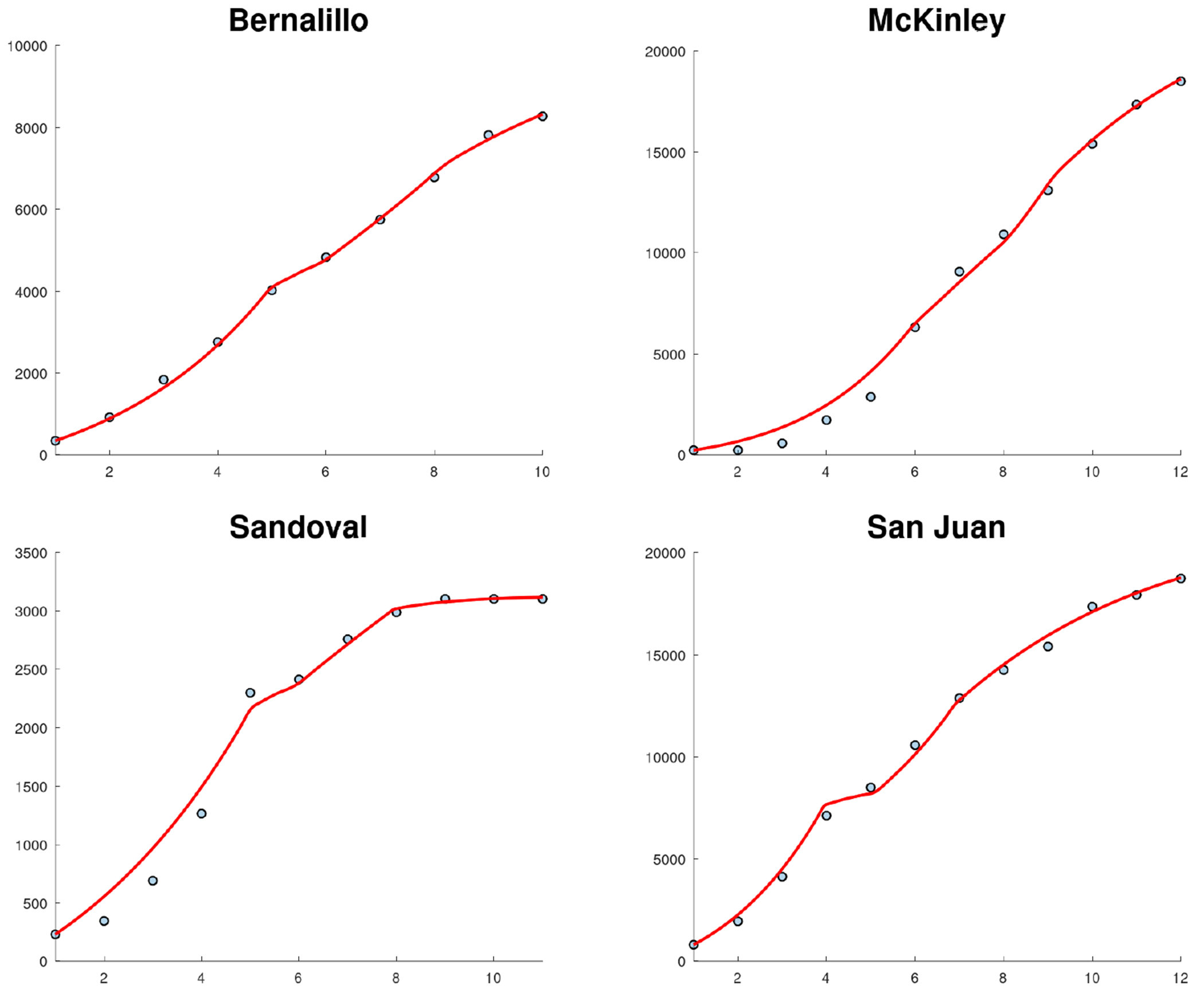
Fits of the SIR model to the cumulative COVID cases for some New Mexico state counties. The fits use different transmission rates due to the implementation of NPIs at different times.

Figure 7 shows the fit of the SIR model to the cumulative cases of COVID in New Mexico using different phases due to the NPIs. The fit is very accurate, despite the fact that the data includes cumulative cases from different counties that all have different characteristics related to population and geography. The fit is also more accurate than previous ones due to the incorporation of different transmission rates in order to reflect the effect of NPIs and spatial-temporal factors. Figure 8 shows the fit of the SIR model to the cumulative COVID cases of some New Mexico counties. The fits use different transmission rates due to the implementation and adoption of NPIs at different times. For instance, notice that the first phase for McKinley County is the longest and ending on April 21. Table 4 presents the estimated transmission rates for each of the phases for some counties. It can be seen that during the first phases all the counties show a basic reproduction number greater than one. These results agree with the increase in COVID cases observed at the beginning of the pandemic in New Mexico. However, during the second phases all the basic reproduction numbers decreased. Moreover, for all counties they were less than one or very close to one. The largest transmission rate is for McKinley County and that is probably related to its long first phase since it was more difficult to control the spread of SARS-CoV-2. The results related to Sandoval County are very interesting, since after the initial phase all the transmission rates are less than one. However, unfortunately, there is a high degree of uncertainty around Sandoval’s β parameter. Recall that Sandoval County is the fourth most populous county in New Mexico and is part of the Albuquerque metropolitan area. In addition, the transmission rates for the contiguous Bernalillo County after the first phase are less than or almost one. Thus, it seems that similar situations occurred in Sandoval and Bernalillo Counties, which are part of the Albuquerque metropolitan area. One possible explanation for this is that the urban population followed COVID-19 pandemic policies more closely than the rural population [73]. In fact, it has been found that the rural population is less likely to follow COVID-19 related policies. In addition to this, there are other reasons that increase the fatalities and cases in rural areas [74–76]. Another important result is that there is a significant decrease in the transmission rate in Phase 4 for all the counties even though the previous phase showed an increase for all of them. One explanation for this result is that during Phase 4 masks for retailers were implemented (May 5) and masks were made mandatory for everyone on May 15th [35]. All these previous results suggest that the implementation of NPIs had an impact on the dynamics of the pandemic and/or the increase in cases created more awareness about the risk of the COVID-19 pandemic in these counties of New Mexico. It is important to note that New Mexico kept the COVID case numbers relatively low due to the NPIs passed by Governor Michelle Grisham [77]. Another factor that could have affected transmission rates was the news related to the COVID-19 pandemic [78].

#### 4.2.2. Fits of the SIR model to new COVID-19 cases

In this section, we present the fits of the SIR model to the new cases of COVID using different transmission rates due to the implementation of a variety of NPIs.

**Figure 9.**
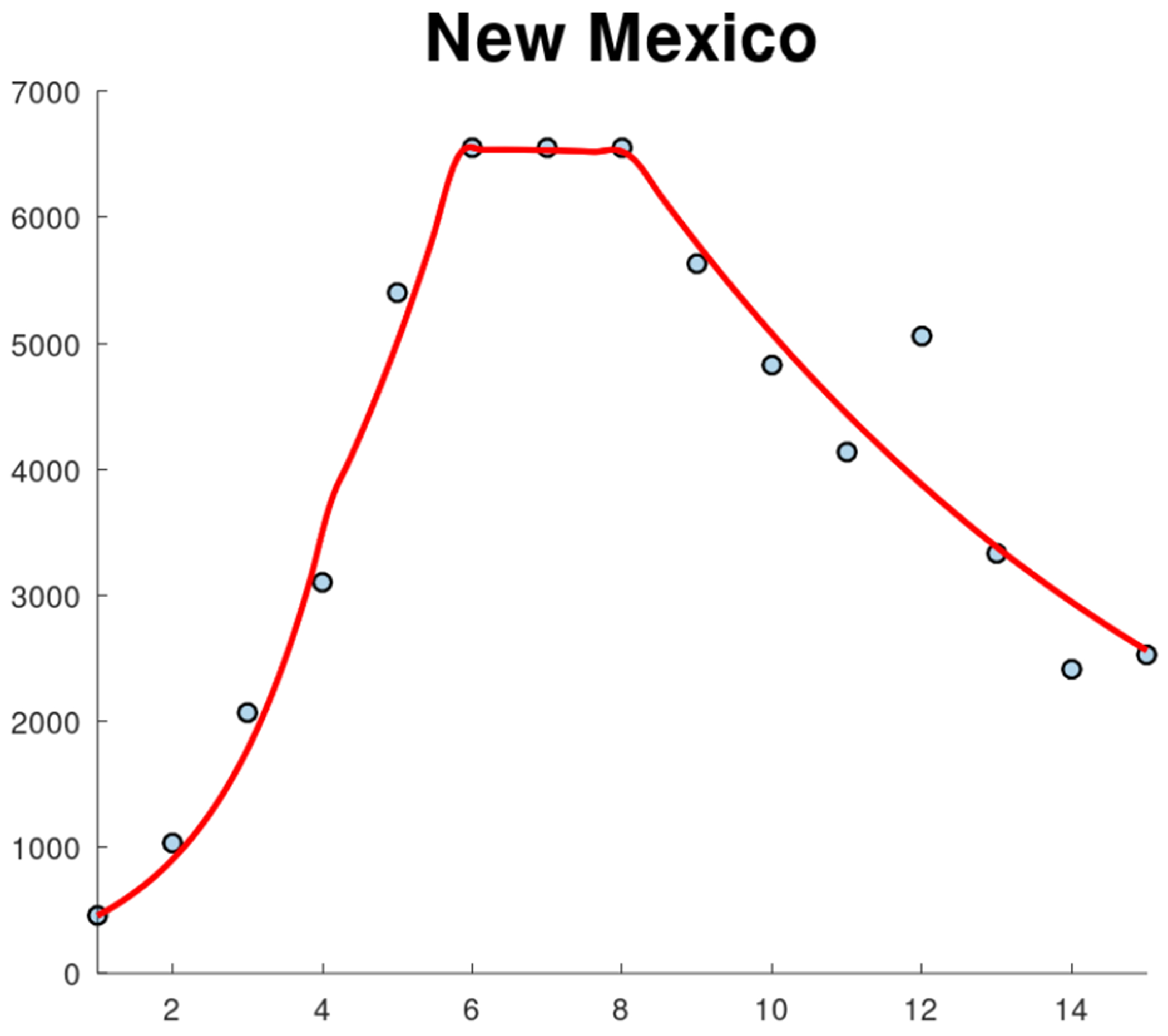
Fits of SIR model (3.4) to the new cases of COVID-19 in New Mexico state using different New Mexico phases.

**Figure 10.**
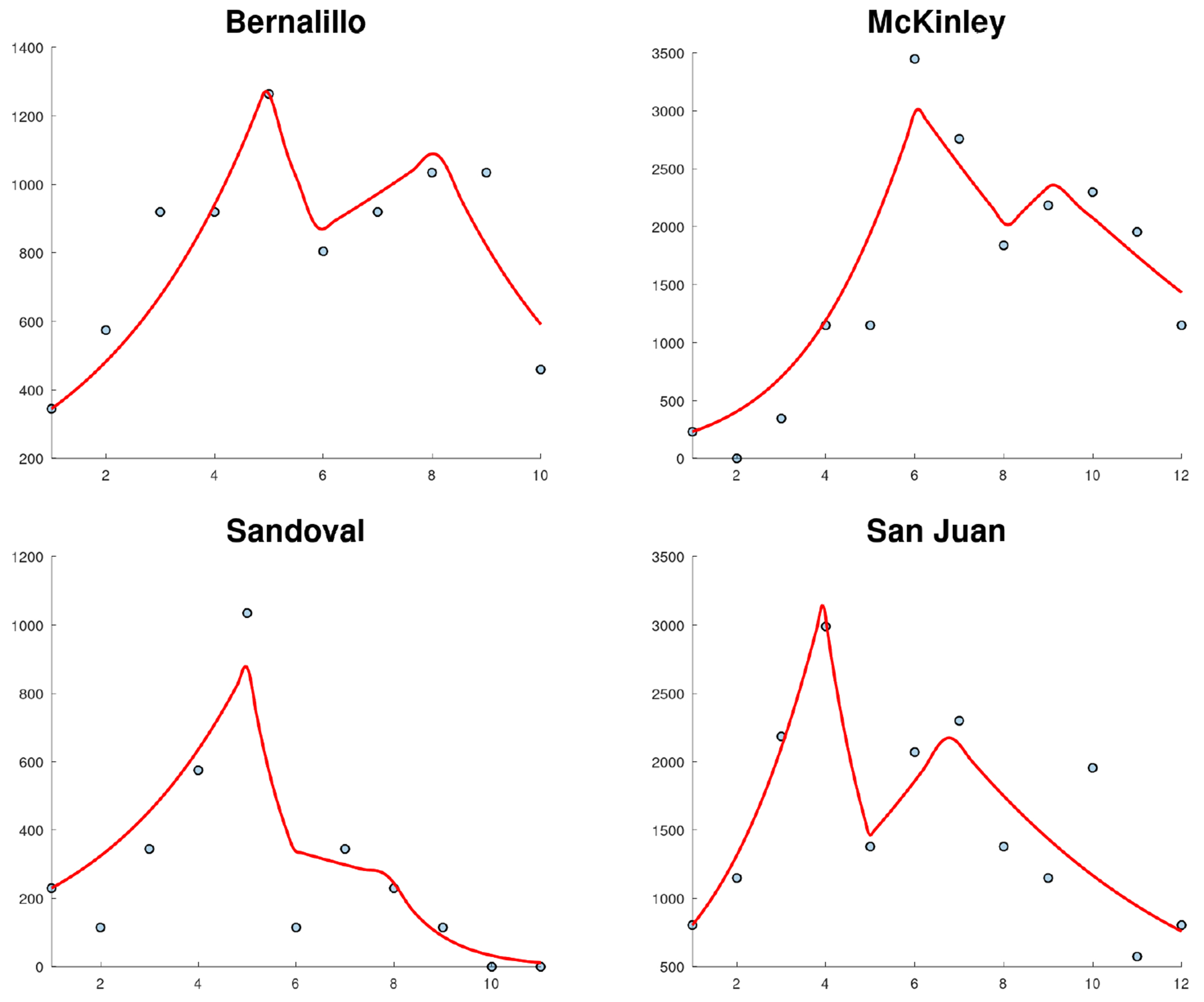
Fits of SIR model (3.4) to the new cases of COVID-19 in some New Mexico state counties. The fits use different transmission rates due to the implementation of NPIs at different times.

Figure 9 shows the fit of the SIR model to the new weekly cases of COVID in New Mexico using different phases due to the NPIs. This fit is also very accurate, despite that in this case the data is for new cases which are more difficult to fit than cumulative cases since the profile of the data increases and then decreases [47, 49]. The fit reflects the effect of changes in human behavior and spatial-temporal factors [52, 75]. Figure 10 shows the fit of the SIR model to the new cases of COVID-19 in some New Mexico counties. As before, the fits use different transmission rates. Again, the fits are relatively accurate considering that the data are for new cases. The fit of the model to Sandoval County shows a monotonous decrease after the first peak, which is different than what is obtained for the other counties. Sandoval County is less populated than the contiguous Bernalillo County. In addition, its population density (14/km^2^) is significantly less than that of Bernalillo ((220/km^2^). Thus, these factors could have affected the spread of SARS-CoV-2 in different ways [10, 30, 78, 79].

With regard to the new cases of COVID in Bernalillo, McKinley, and San Juan Counties, it can be seen in Figure 10 that they have very similar patterns with four clear phases with different transmission rates. This strongly suggests that changes in human behavior occurred due to the implementation of NPIs and changes in people’s awareness of the COVID-19 pandemic [10, 30, 78, 79]. These results also agree with previous results in the scientific literature. For example, in [2] a time-varying vulnerability index was proposed for the state of New Mexico to identify which communities had a higher risk of negative outcomes from the COVID-19 pandemic. The vulnerabilities of the counties was found to change during the early phase of the COVID-19 pandemic.

Table 5 presents the estimated transmission rates for each of the phases and for the different counties. It can be seen that during the first phases all the counties show a basic reproduction number greater than one. These results agree with the increase in COVID cases observed at the beginning of the pandemic in New Mexico. During the counties’ second phase all the basic reproduction numbers decreased, and they were less than one. This suggests that the implementation of NPIs had an impact on the dynamics of the pandemic and/or the increase in cases created more awareness about the risk of the COVID-19 pandemic in the population. Thus, regardless of the impact of the NPIs it is clear that the human behavior of the New Mexico population changed during the early phase of the COVID-19 pandemic. Note that the introduction of new SARS-CoV-2 variants during the early phase was unlikely or small [80].

## 5. Discussion

In this paper, we used empirical and mechanistic ODE models to examine the early phase of the COVID-19 pandemic in the state of New Mexico, USA, and in particular, in the most populous counties of New Mexico. The exponential growth model provided results with regard to the basic reproduction number ${ℛ}_{0}$ and the exponential growth rate r. The estimation of these numbers partially agrees with the results presented in other works [18]. However, it is important to note that the estimates for the basic reproduction number ${ℛ}_{0}$ and the effective reproduction number ${ℛ}_{t}$ from New Mexico are lower than those presented in other works for the USA. For example, in [18] a systematic review of the basic reproduction number was presented. A mean of 3.38 was found for ${ℛ}_{0}$ with a range of 1.90 to 6.49. In [81] it was found that in the U.S., ${ℛ}_{0}$ decreased monotonically after March 20, and then fell below 2 on April 6. From May 18 to July 29 it oscillated between 1.0 and 1.1. In [23] estimates of ${ℛ}_{0}$ in ranges from 7.1 to 2.3 were reported for different states in the USA. The lower values of New Mexico counties could be due to effective NPIs, lower population density, and greater awareness of the COVID disease. In New Mexico there was a variety of NPIs used as well as changes to them throughout the pandemic [35].

With regard to the SIR model based on a system of differential equations, we used new weekly COVID cases and also cumulative cases to estimate transmission rates in different phases of the pandemic for some New Mexico counties. We carefully selected these counties, as they were the only ones with enough deaths to use to estimate cases. It is important to note that death data is often more reliable than reported cases which are affected by many factors [61, 82, 83]. We found that for the beginning of the early phase of the COVID-19 pandemic in New Mexico, the most populous counties had a basic reproduction number greater than one, but later in some counties ${ℛ}_{0}$ dropped below one. Due to this significant change, we implemented a time-varying transmission rate in order to better characterize the dynamics of the spread of SARS-CoV-2 during the early phase of the pandemic. Few works have used time-varying transmission rates for mathematical modeling of the COVID-19 pandemic [56, 64, 84–86]. This is necessary when significant changes occur in the trends in the number of COVID cases. Other works have found large differences in the value of ${ℛ}_{0}$ between the calibrated SEIRD and SIR models. However, both models provided goodness of fit to the data [87].

The first wave of the COVID-19 pandemic was particularly severe in McKinley, Sandoval, San Juan, and Bernalillo Counties. However, there are marked differences in the course the early pandemic took in these counties. In Bernalillo, Sandoval, and San Juan Counties, the NPIs appear to have been successful in lessening the severity of the pandemic. For these counties, the effective reproduction number ${ℛ}_{t}$ in the second phase of the pandemic in New Mexico was less than one, indicating that the epidemic was being reduced or contained at this time. This is supported by the results of the SIR model, which showed that the estimated transmission rates decreased for the later phases, indicating that the spread of SARS-CoV-2 was reduced. Public police changes appear to have been particularly effective in lessening the severity of the pandemic in Sandoval County, which started the pandemic with a high ${ℛ}_{0}$, but showed an extreme drop in the spread of SARS-CoV-2 in the second phase. The effectiveness of New Mexico’s public police changes or people’s awareness is further reflected in other counties we examined, where the transmission rate decreased in the second phase and other phases. This suggests that NPIs had some impact in reducing the burden of the pandemic and that people’s behavior changed during these early phases. However, NPIs appear to have been less effective in decreasing the severity of the COVID-19 pandemic in McKinley and San Juan counties. For example, the highest transmission rate was obtained for McKinley County and also the longest first phase. This could be related to a greater difficulty in implementing NPIs to control the spread of SARS-CoV-2. The racial makeup of McKinley County is approximately 75% Native American, and this also could be a factor when implementing NPIs. McKinley and San Juan Counties had effective reproduction numbers well more than one in the third phase and higher transmission rate values. In particular, both counties contain portions of the Navajo Nation. The prevalence of COVID cases in the Navajo Nation was among the highest in the United States. There was a significant increase in the number of cases during the early phase of the COVID-19 pandemic with a doubling time of 10.12 days [34]. In addition, McKinley County has San Juan as an adjacent county, and thus a similar pattern of the COVID-19 pandemic may be expected. San Juan County showed a similar pattern, but did experience a significant increase in cases around the end of June that corresponds to the statewide second wave. On the other hand, Sandoval County had a different pattern in the early phase of the COVID-19 pandemic from that of McKinley County and San Juan County. This could be due to the fact that Sandoval County is adjacent to Bernalillo County, which is the most populous county in New Mexico. Therefore, the mobility of people could have affected the spread of SARS-CoV-2 in a different way than McKinley and San Juan Counties. In summary, in this study we have seen that the dynamics of the COVID-19 pandemic were different in New Mexico counties. These differences can be explained using different spatial-temporal and cultural factors. Nevertheless, we have shown a variety of results that strongly suggest that NPIs and people’s awareness of the pandemic played a role in the dynamics of the COVID-19 pandemic in New Mexico. Finally, it is important to mention that, as with any work that includes mathematical modeling, there are limitations. In the models used in this work, we have not explicitly included asymptomatic cases. The viral load in asymptomatic people has been found to be similar to that in symptomatic people [88]. Another complex problem related to asymptomatic people is unreported cases. In our work, we have used death data in order to deal with the issue of unreported cases, but there are other approaches [61, 82, 89, 90]. Despite these limitations, we have provided deeper insight into the characterization of the dynamics of the early phase of the COVID-19 pandemic in New Mexico, USA. Furthermore, we have presented several features related to the spread of SARS-CoV-2 and the impact of the implementation of NPIs. With regard to estimated parameters, these can be used to better understand the geographical spread of the disease looking at social and other indicators [91]. In particular, in [91] the authors studied how the parameters are influenced by uneven development, human movement, and trade differences. In our work, we showed that geographical factors and therefore social factors (due to differences between the counties’ populations) can influence the spread of SARS-CoV-2 and other viruses.

## 6. Conclusions

In this paper, we used a variety of mathematical models to characterize the early phase of the COVID-19 pandemic in the state of New Mexico. We used both empirical and mechanistic ODE models to examine the dynamics of the pandemic in New Mexico and in carefully selected New Mexico counties. To the best of our knowledge, mathematical models have not yet been used to characterize and explain the crucial early phase of the COVID-19 pandemic in New Mexico. Thus, this work provides information on the dynamics of the COVID-19 pandemic that can be useful for public health and can offer advice for future epidemics. With regard to the empirical model, we used the exponential growth model. This model was used to estimate the exponential growth rate and the basic reproduction number ${ℛ}_{0}$. In addition, we used the SIR model to estimate ${ℛ}_{0}$ using new weekly COVID cases and also cumulative cases. We found that for the beginning of the early phase of the pandemic, the most populous counties had a basic reproduction number greater than one. Transmission rates were found to vary significantly during the early phase of the pandemic. Moreover, ${ℛ}_{0}$ dropped below one during some phases for some counties. This suggests that NPIs had some impact in reducing the burden of the pandemic and that people’s behavior changed during this early phase. Future studies that address this important health topic can explore these factors in more detail if data are available.

## Figures and Tables

**Table 1.** Estimated parameters with 95% confidence intervals estimated by using Gaussian error structure for the exponential growth model (3.1) and the SSR for the most densely populated counties in New Mexico state. The calibration is for the first wave (peak) during the early phase of the COVID-19 pandemic.

County	*r*	*R*_0_	*S S R*
State Wide	0.58 (0.53–0.62)	1.58 (1.53–1.62)	3.8463e+05
Bernalillo	0.85 (0.83–0.87)	1.85 (1.83–1.87)	1.0229e+02
Chaves	0.92 (0.66–1.08)	1.92 (1.66–2.08)	3.3101e0
Curry	0.51 (0.38–0.6)	1.51 (1.38–1.6)	2.1861e0
Dona Ana	0.48 (0.46–0.5)	1.48 (1.46–1.5)	3.3068e+03
Eddy	0.18 (0.14–0.21)	1.18 (1.14–1.21)	9.1953e+01
Lea	0.26 (0.26–0.27)	1.26 (1.26–1.27)	1.3521e+03
Luna	0.2 (0.19–0.21)	1.2 (1.19–1.21)	5.5820e+03
McKinley	0.9 (0.84–0.94)	1.9 (1.84–1.94)	7.0236e+04
Otero	0.26 (0.17–0.31)	1.26 (1.17–1.31)	5.1277e+01
Rio Arriba	0.23 (0.21–0.25)	1.23 (1.21–1.25)	4.1849e+03
Roosevelt	0.25 (0.16–0.29)	1.25 (1.16–1.29)	1.0687e+02
San Juan	0.75 (0.7–0.78)	1.75 (1.7–1.78)	4.3551e+04
Sandoval	1.04 (0.94–1.12)	2.04 (1.94–2.12)	3.0528e+03
Santa Fe	0.97 (0.96–0.99)	1.97 (1.96–1.99)	3.2816e-01
Taos	1.03 (0.96–1.09)	2.03 (1.96–2.09)	6.6652e-01
Valencia	0.54 (0.46–0.59)	1.54 (1.46–1.59)	3.9661e-01

**Table 2.** Estimated parameters for the exponential growth model (3.1) and SSR for the most densely populated counties in New Mexico state. The calibration is for the second and often highest peak over the first 25 weeks.

County	*r*	*R*_0_	*S S R*
Bernalillo	0.41 (0.34–0.46)	1.41 (1.34–1.46)	2.61763E+04
Chaves	0.17 ( 0.12–0.2)	1.17 (1.12–1.2)	1.37187E+02
Curry	0.28 (0.23–0.32)	1.28 (1.23–1.32)	3.81888E+01
Dona Ana	0.34 (0.33–0.35)	1.34 (1.33–1.35)	2.57031E+04
Eddy	0.21 (0.2–0.22)	1.21 (1.2–1.22)	2.89149E+02
Lea	0.26 (0.26–0.27)	1.26 (1.26–1.27)	1.35218E+03
Luna	0.2 (0.19–0.21)	1.2 (1.19–1.21)	5.58199E+02
McKinley	0.9 (0.85–0.94)	1.9 (1.85–1.94)	7.02356E+04
Otero	0.18 (0.16–0.19)	1.18 (1.16–1.19)	2.90195E+02
Rio Arriba	0.23 (0.21–0.25)	1.23 (1.21–1.25)	4.18490E+03
Roosevelt	0.16 (0.13–0.17)	1.16 (1.13–1.17)	4.60109E+02
San Juan	0.75 (0.71–0.79)	1.75 (1.71–1.79)	4.35511E+04
Sandoval	0.21 (0.17–0.24)	1.21 (1.17–1.24)	3.47826E+04
Santa Fe	0.16 (0.15–0.17)	1.16 (1.15–1.17)	2.18186E+03
Taos	0.13 (0.12–0.14)	1.13 (1.12–1.14)	1.28828E+02
Valencia	0.22 (0.22–0.23)	1.22 (1.22–1.23)	8.58807E+02

**Table 3.** Phases considered for the different transmission rates due to implementation and adoption of NPIs in some counties of New Mexico [35].

County	Phase 1	Phase 2	Phase 3	Phase 4
New Mexico	Mar 10–Mar 31	Apr 1–Apr 15	Apr 16–Apr 30	May 1–June 19
Bernalillo	Mar 10–Apr 7	Apr 8–Apr 15	Apr 16–Apr 30	May 1–May 15
McKinley	Mar 17–Apr 21	Apr 22–May 7	May 8–May 14	May 15–June 5
Sandoval	Mar 17–Apr 14	Apr 15–Apr 22	Apr 23–May 7	May 8–May 22
San Juan	Mar 24–Apr 14	Apr 15–Apr 22	Apr 23–May 7	May 8–June 12

**Table 4.** Fits of the SIR model (3.4) to the cumulative cases using different phases for each county.

County	*β*_1_	*β*_2_	*β*_3_	*β*_4_	RSS
New Mexico	1.65 (1.47–1.74)	1.29 (1.09–1.51)	1.15 (1.03–1.53)	0.88 (0.76–0.91)	3.6367e+06
Bernalillo	1.33 (1.28–1.37)	0.65 (0.06–0.84)	1.1 (1.01–1.36)	0.78 (0.61–0.95)	7.6032e+04
McKinley	1.49 (1.31–1.62)	1.12 (0.52–1.51)	1.52 (1.04–2.33)	1.05 (0.69–1.35)	3.56748e+06
Sandoval	1.25 (1.07–1.36)	0.46 (0–1.34)	0.97 (0.18–1.32)	0.27 (0–1.1)	1.9937e+05
San Juan	1.47 (1.32–1.56)	0.3 (0–0.89)	1.46 (1.1–1.6)	0.92 (0.74–1.04)	1.2985e+06

**Table 5.** Fits of SIR model (3.4) to the new cases using different phases for each county.

County	*β*_1_	*β*_2_	*β*_3_	*β*_4_	RSS
New Mexico	1.68 (1.63–1.78)	1.33 (1.09–1.42)	1.01 (0.93–1.16)	0.89 (0.84– 0.92)	2.2742e+06
Bernalillo	1.34 (1.28–1.38)	0.59 (0.17–0.99)	1.12 (0.89–1.33)	0.68 (0.4–0.96)	1.4202+05
McKinley	1.58 (1.52–1.63)	0.95 (0.63–1.2)	1.46 (0.85–2.03)	1.11 (0.78–1.38)	1.4264e+06
Sandoval	1.35 (0.02–1.41)	0 (0–0.98)	0.89 (0–3.58)	0 (0–1.39)	1.3883e+05 1
San Juan	1.51 (1.41–1.57)	0.28 (0– 0.79)	1.35 (1.04–1.52)	0.92 (0.71–1.05)	1.0946e+06

**Figure A1.**
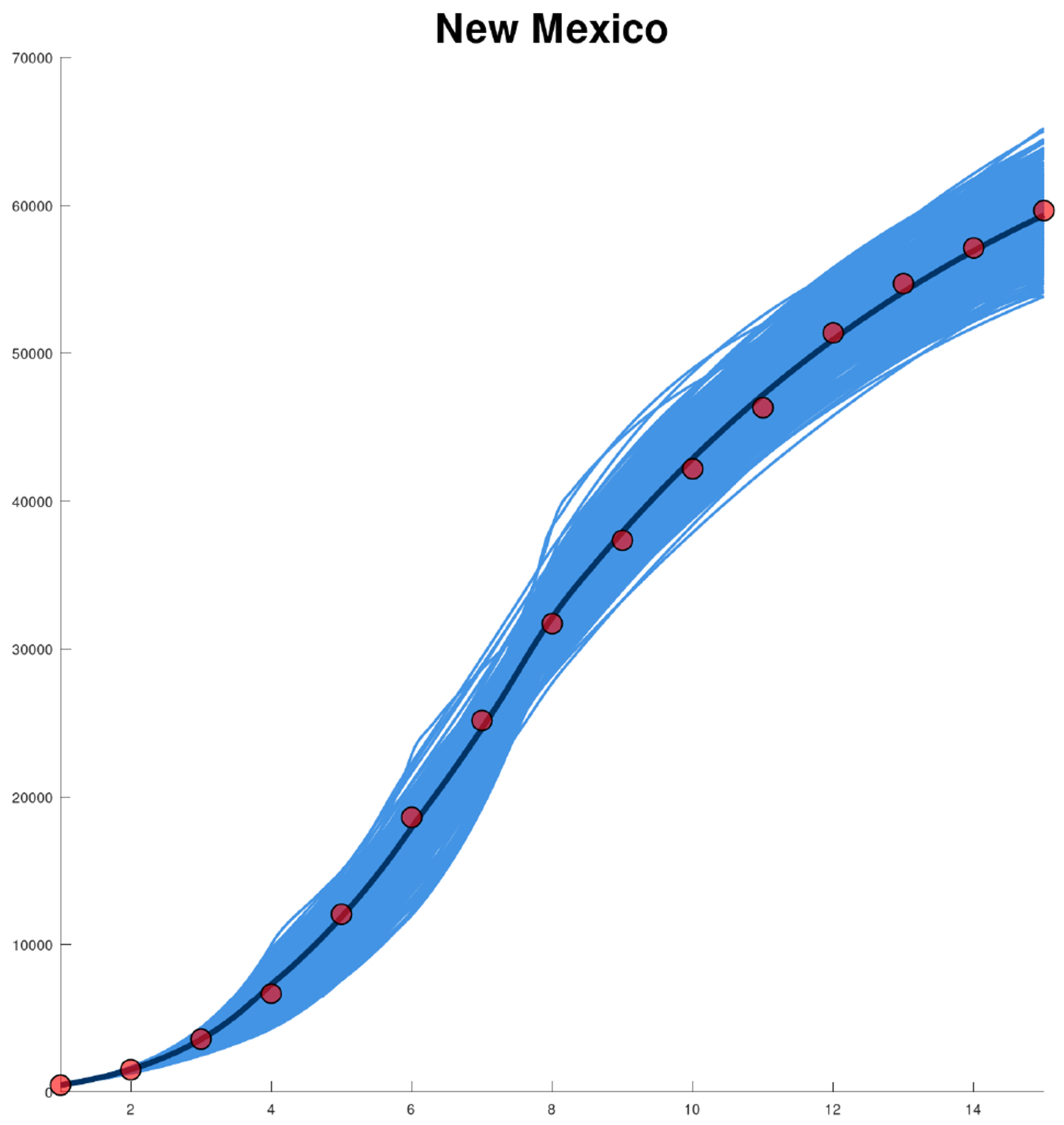
Bootstrapping fits of the SIR model to the cumulative COVID cases of New Mexico state. The fits use different transmission rates due to the implementation of NPIs at different times. The bootstrap results are shown in blue, the model’s best fit in black, and the cumulative cases in red points.

**Figure A2.**
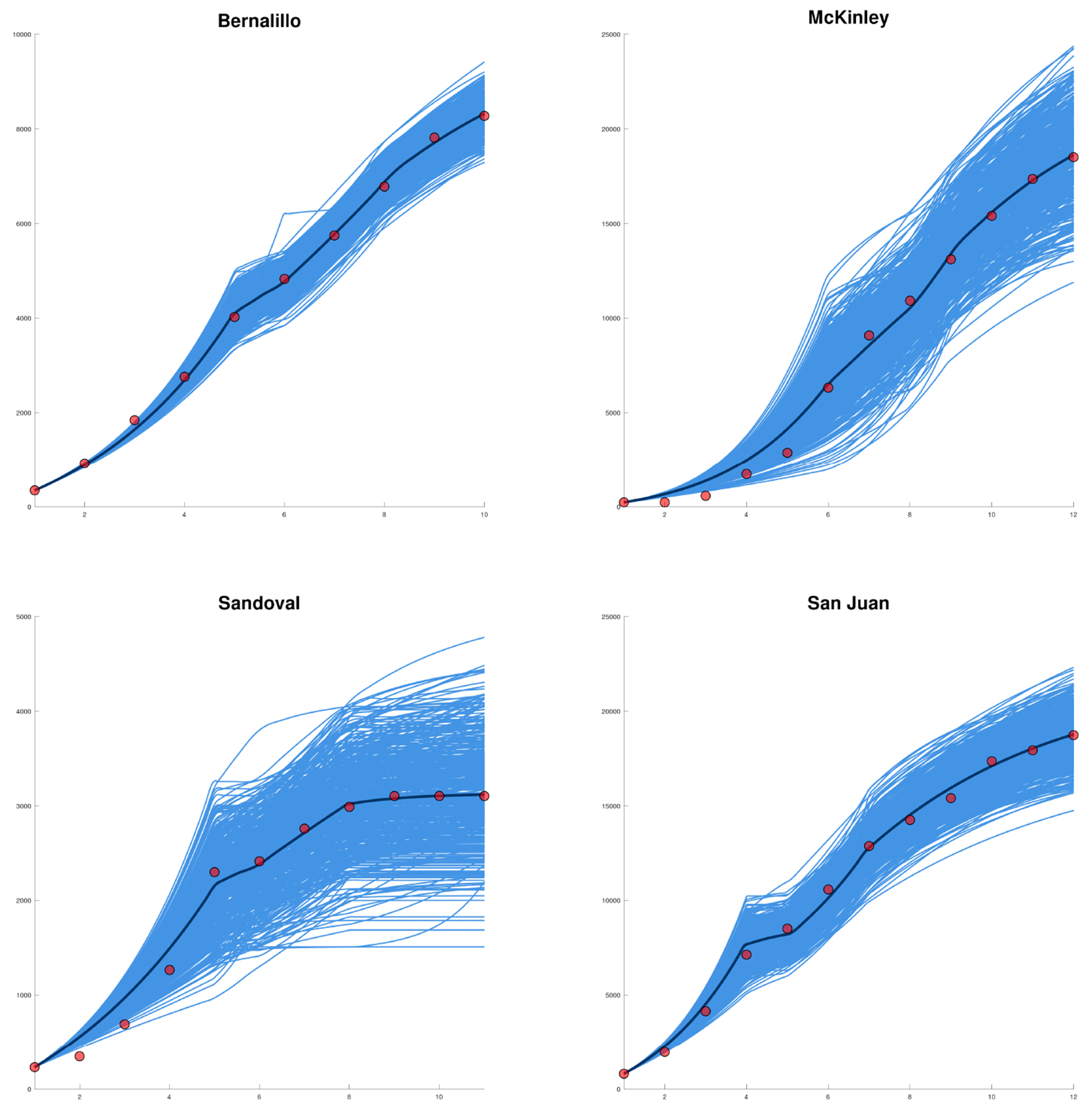
Bootstrapping fits of the SIR model to the cumulative COVID cases in some New Mexico state counties. The bootstrap results are shown in blue, the model’s best fit in black, and the cumulative cases in red points.

**Figure A3.**
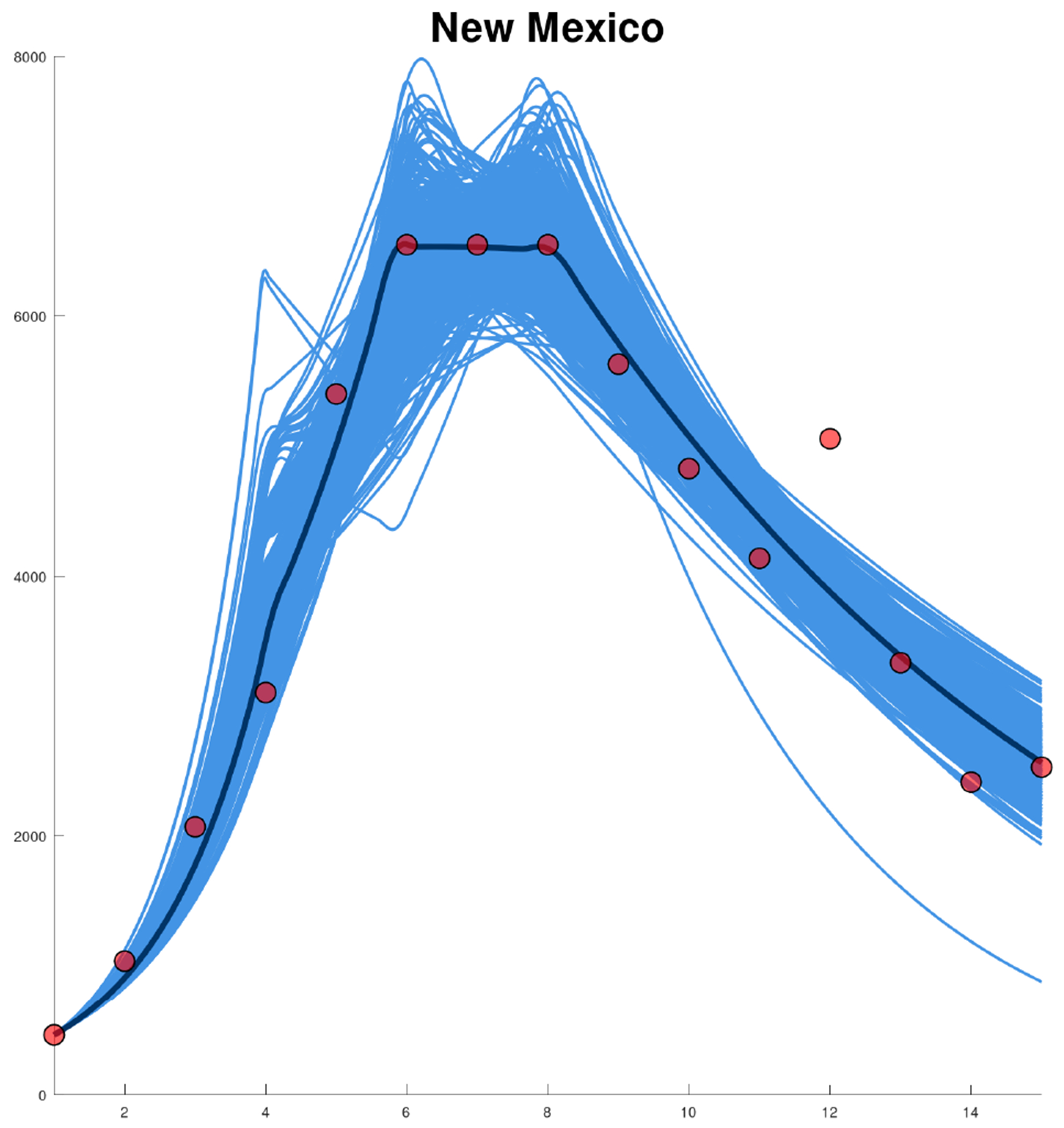
Bootstrapping fits of the SIR model to the incidence of COVID cases in New Mexico. The bootstrap results are shown in blue, the model’s best fit in black, and the cumulative cases in red points.

**Figure A4.**
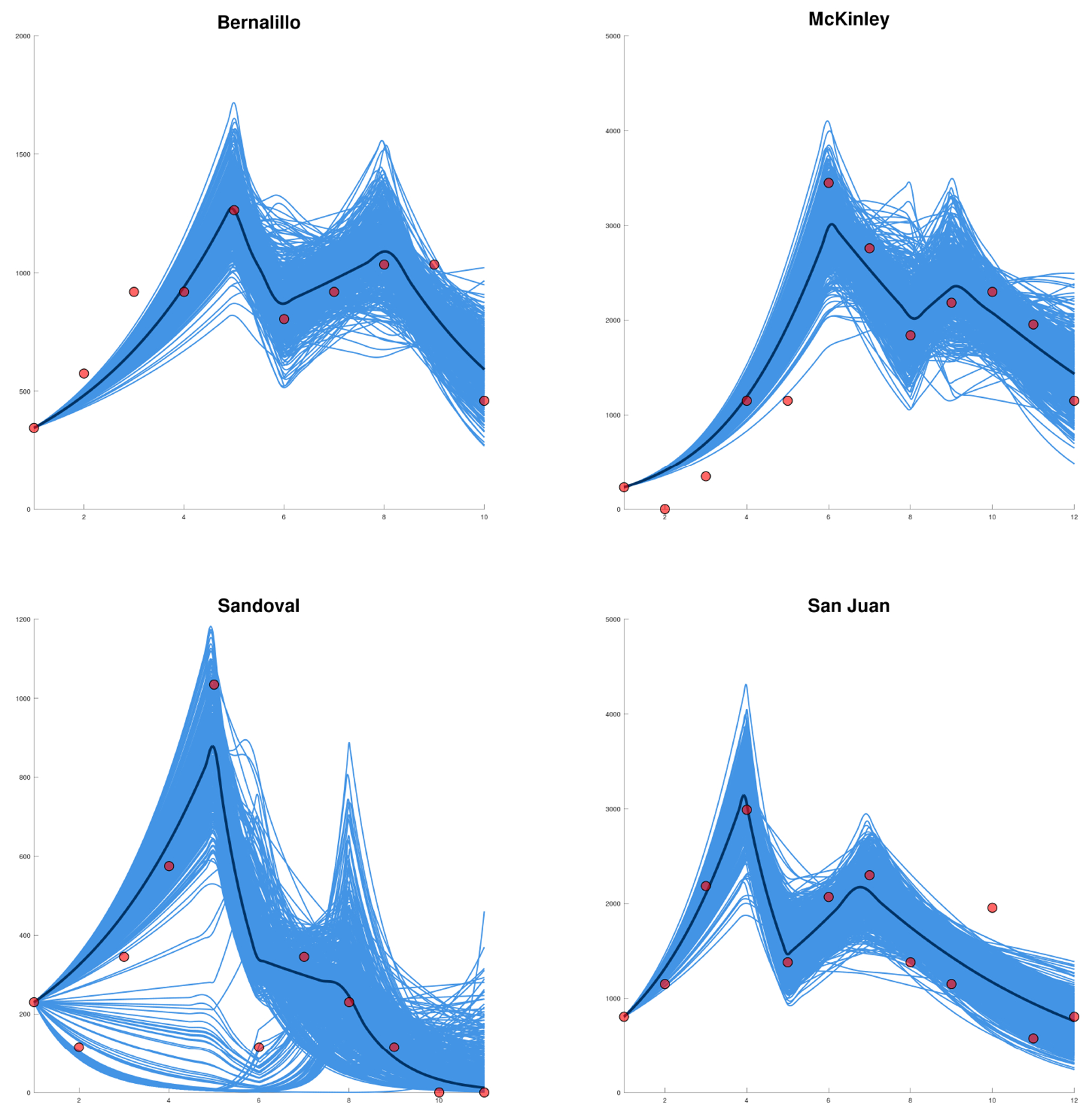
Bootstrapping fits of the SIR model to the incidence of COVID cases in some New Mexico state counties. The bootstrap results are shown in blue, the model’s best fit in black, and the cumulative cases in red points.

## Appendix

In this section, we provide several bootstrapping fits of the SIR model to cumulative COVID cases and incidence.

### Bootstrapping fits of the SIR model to cumulative COVID cases

Figure A1 shows the bootstrap fits of the SIR model to the cumulative COVID cases in New Mexico. Figure A2 shows the bootstrap fits of the SIR model to the cumulative COVID cases in some New Mexico counties.

### Fits of the SIR model to the incidence of COVID-19 cases

Figure A3 shows the bootstrap fits of the SIR model to the incidence of COVID cases in New Mexico. Figure A4 shows the bootstrap fits of the SIR model to the incidence of COVID cases in some New Mexico counties.
